# From past to future: Bibliometric analysis of global research productivity on nomogram (2000–2021)

**DOI:** 10.3389/fpubh.2022.997713

**Published:** 2022-09-20

**Authors:** Xiaoxue Wang, Jingliang Lu, Zixuan Song, Yangzi Zhou, Tong Liu, Dandan Zhang

**Affiliations:** ^1^Department of Health Management, Shengjing Hospital of China Medical University, Shenyang, China; ^2^Lanzhou Information Center, Chinese Academy of Sciences, Lanzhou, China; ^3^Department of Obstetrics and Gynecology, Shengjing Hospital of China Medical University, Shenyang, China

**Keywords:** bibliometric analysis, Bibliometrix, Biblioshiny, nomogram, data mining

## Abstract

**Background:**

Nomogram, a visual clinical predictive model, provides a scientific basis for clinical decision making. Herein, we investigated 20 years of nomogram research responses, focusing on current and future trends and analytical challenges.

**Methods:**

We mined data of scientific literature from the Core Collection of Web of Science, searching for the original articles with title “Nomogram^*^/Parton Table^*^/Parton Nomogram^*^”, published within January 1st, 2000 to December 30th, 2021. Data records were validated using HistCite Version and analyzed with a transformable statistical method, the Bibliometrix 3.0 package of R Studio.

**Results:**

In total, 4,176 original articles written by 19,158 authors were included from 915 sources. Annually, Nomogram publications are continually produced, which have rapidly grown since 2018. China published the most articles; however, its total citations ranked second after the United States. Both total citations and average article citations in the United States rank first globally, and a high degree of cooperation exists between countries. Frontiers in Oncology published the most papers (238); this number has grown rapidly since 2019. Journal of Urology had the highest H-index, with an average increase in publications over the past 20 years. Most research topics were tumor-related, among which tumor risk prediction and prognostic evaluation were the main contents. Research on prognostic assessment is more published and advanced, while risk prediction and diagnosis have good developmental prospects. Furthermore, nomogram of the urinary system has been highly developed. Following advancements in nomogram modeling, it has recently been applied to non-oncological subjects.

**Conclusion:**

This bibliometric analysis provides a comprehensive overview of the current nomogram status, which could enable better understanding of its development over the years, and provide global researchers a comprehensive analysis and structured information to help identify hot spots and gaps in future research.

## Introduction

With development of biostatistics methodology and the increasing dependence of clinical research on statistical methods, increasingly more statistical predictive models have been widely used in various aspects of clinical research, such as screening of influencing factors, diagnostic tests, prognostic evaluation, and individualized treatment (1, 2). Among many prediction models, nomogram, with its unique advantages, is being used by more medical researchers. Nomogram is a graphical tool that can quickly approximate complex calculations without the need for a computer or calculator; a cluster of non-intersecting line segments is used to represent the functional relationship between multiple variables in plane coordinates. Its advantage is that the value of a variable, such as the patient's index score or survival probability, can be directly calculated using the graph. This nomogram primarily visualizes the results of the regression equation, which is usually used for logistic regression or COX regression to draw multiple line segments in a specific proportion based on the regression results, so that the risk of disease or survival probability of an individual can be easily calculated.

This nomogram invented in the 19th century has been used in medicine since then, including the percentile and probabilistic nomograms (3, 4). Percentile nomograms are used to determine the percentile of an individual's measurement value in the population; probabilistic nomograms would be used to determine the probability of an individual specific event, such as occurrence, recurrence and prognosis (such as death), based on multivariate dichotomous regression or a COX proportional risk model.

The advantages of nomogram over traditional prediction models are as follows: first, the simple and straightforward nomogram prediction model can be applied to clinical decision-making more easily than complex statistical models. Interpreting a traditional statistical model requires professional statistical knowledge; furthermore, clinicians and patients find it difficult to understand its practical significance, thus limiting its practical application. The nomogram prediction model can be effectively applied in clinical practice, and improves doctor-patient communication by predicting patient outcomes and presenting complex statistical models and related risk variables with risk scores. Second, the nomogram prediction model would be able to predict clinical outcomes of an individual based on individual characteristics, thus promoting personalized treatment. For example, modern medical oncology generally uses the TNM staging, proposed by Pierre Denoix et al. (in 1943), to stage a patient's tumor and predict prognosis (5). The biggest flaw of the TNM staging is that it only determines the anatomical grade of the patient's tumor, and cannot take into account the actual situation of the patient, such as age, gender, race, complications, laboratory examination indicators, which has certain limitations in individual prediction. This nomogram prediction model can integrate more prognostic factors in combination with actual patient and tumor characteristics, thus providing a more accurate prediction of individual prognosis (1). At present, increasingly more literatures have confirmed the higher value of nomogram in the diagnosis and prognosis of various tumors, and it is replacing the traditional staging system as the new standard for tumor diagnosis and prognosis.

Since Puppo and Perachino first applied the nomogram model to predict lymph node metastasis of prostate cancer in 1997, the nomogram model has been widely used for clinical prediction and decision making (6). The standard procedures for establishing this clinical nomogram prediction model are as follows: first, establish the purpose and question of the study; determine outcomes, such as diagnosis, disease-free survival and overall survival according to the purpose; and collect data from the study population. Secondly, establish the prediction model by selecting a model according to the research purpose; screen out and end contact real prediction variables, and variables related to processing (such as consideration of the interaction between variables, the inspection result of linear relationship between risk and variable, and so on); then, estimate the coefficient model and consider the shrinkage coefficient; finally evaluate and validate the model.

With the advent of digital age, nomograms may no longer be popular and required. Currently, we are able to perform very complex calculations more quickly, and high-performance computers are becoming portable and more accessible. Even a smartphone app can provide faster and more accurate calculations than a nomogram. However, overly complex models often make them difficult to interpret, which discourages clinicians from using them in practice. Nomograms can create an online risk calculator to explain the impact of each prediction in more detail by graphically presenting the effect of each prediction. Diagrams like nomogram would be better for spreading knowledge and improving health care, causing more articles to use nomograms to relay their findings.

Bibliometric analysis is a unique concept first proposed by Pritchard in 1969 (7). In recent years, due to the increase in the number of publications used for analysis, and the popularity of user-friendly computer analysis programs, bibliometric analysis has become a widely used research method (8–10). However, no bibliometric studies on nomograms exist. In view of the need to further understand the status of nomogram research, this bibliometric study aimed to provide an updated overview over the period of 2000–2021.

## Methods

### Source of bibliometric data and search strategy

We used the Web of Science (WOS) (Clarivate Analytics, Philadelphia, PA, USA) database to identify nomogram research articles. Science Citation Index Expanded (SCIE) and Social Science Citation Index (SSCI) were selected in the WOS Core Collection online database. The data of WOS comes from journals, books, patents, conference proceedings, network resources (including free and open resources), etc. The platform's three citation index systems SCIE, SSCI, Arts & Humanities Citation Index (A&HCI) include more than 12400 authoritative and high-impact international academic journals worldwide. SCIE has always been recognized as the most authoritative scientific and technological literature retrieval tool by the global academic community, providing the most important research information in the field of science and technology. A total of more than 8,600 world authoritative journals in the field of natural sciences are included, covering 176 disciplines. SSCI is a comprehensive multi-disciplinary database covering the field of social sciences, including more than 3,000 world authoritative journals in the field of social sciences, covering 56 subject areas.

The following search strategies were adopted in this study: (((TI = (nomogram^*^)) OR TI = (parton Table^*^)) OR TI = (parton Nomogram^*^)) AND DT = (Article), publication time: 2000-01-01 to 2021-12-30. The search took place on March 10, 2022. Only original articles (based on the WOS field label PT) were included in the bibliometric analysis.

### Bibliometric analysis

Using HistCite version 12.03.17 (11), we first checked for anomalies in the qualifying records retrieved from the WOS. Biblioshiny, a graphical web interface based on Bibliometrix 3.0 [Naples, Italy (12)], was run in the RStudio (https://RStudio.com) environment to analyze the final records.

### Bibliometric measurement

Metadata included print characteristics, such as author name, authors collaboration, total number of publications, total number of citations (TC), journal source, key words, country/region, single/multiple country publications (SCP/MCP), and author-level/source-level indicators, such as h-, m-, and g- indicators. Local Citation (LC) refers to the number of references in this nomogram search database, and Global Citation (GC) refers to the total number of citations of the literature in the WOS database The “Keywords Plus” were semi-automatically assigned by the WOS editorial team based on article titles.

The H-index, a common measure of individual scientific output, is defined as the number of papers with a citation number ≥ h (at least one citation) (13). Thus, it depends on the number of publications a scientist has and the number of citations they receive. The m-index is the h-index divided by the number of years since the author first published a paper (m quotient = h-index/*n, n* = years), reflecting the author's career span. The g-index scores the most cited papers in the dataset to account for the citation evolution of the most frequently cited papers over time. The annual growth rate of scientific publications was assessed using a calculator from www.investopedia.com/calculator/cagr.aspx.

The core source is determined by Bradford's law and can be divided into three regions. Zone 1 is highly productive and considered a nuclear zone. Top countries and institutions are ranked by frequency of publication and total citations. Relationships between affiliation, country, and source were summarized by a Sankey plot (three-fields plot).

Factorial analysis was used to create a conceptual structure map with multiple correspondence analysis (MCA). MCA is a descriptive method for evaluating simple two-dimensional and multiplexed tables containing corresponding metrics between rows and columns, closely grouping indicator levels with similar characteristics; they were well indicated in a 2-dimensional plot forming points clouds. The closer the keywords are to each other, the more related they are. Similarly, hierarchical clustering was used to cluster keywords with the highest similarity to generate a tree graph describing the correlation and de-correlation between keywords in detail.

Thematic maps were used to analyze the importance and development of research topics. Applying the top 250 keywords, entries displayed in the cluster were set to a minimum frequency of 5 in the “Biblioshiny” web software. The number of representative labels for each topic was set to 5. Thematic maps were divided into four quadrants based on density (Y-axis) and centrality (X-axis). Centrality measures the importance of the themes, and density measures the development of the themes. Themes located in the Motor themes quadrant have been developed and form important pillars that form the field of study. The themes reflecting highly developed which located in the Niche themes quadrant. The themes in the emerging or declining themes quadrant are both weakly developed and marginal in the research field. Basic themes quadrants, located at the lower right of the theme map, represent a with higher values of centrality and lower values of density, which are weakly developed but important in the research field.

Sankey diagram was used to demonstrate the evolution of topics, describing in detail the classification, evolution, and correlation of research topics in different periods, and showing the developmental trend and evolutionary route of nomogram, as well as the evolutionary drift points of theme content, intensity, and structure of research fields. Topic evolution was analyzed using Keywords Plus. Each node in the diagram represents a topic, and the node size is proportional to the number of keywords contained in the topic. The linear relationship between nodes represents the evolutionary focus of the research topic, and width indicates the number of shared keywords. The more comprehensive the line, the greater the importance of the two themes.

## Results

From 2000 to 2021, 4,176 original articles on nomogram were cataloged in the WOS Core Collections online database. The articles were derived from 915 sources, using 6,021 keywords plus and 5,721 author's keywords. A total of 19,158 authors contributed to the writing. The collaboration index was 4.46, indicating a high degree of collaboration among the publications. The document-to-author ratio was 0.225, meaning that on an average, ~4–5 authors have collaborated on a document (Table 1).

**Table 1:** Main data information.

**Description**	**Results**
Timespan	2000:2021
Sources (Journals, Books, etc.)	915
Documents	4316
Average years from publication	4.55
Average citations per documents	15.45
Average citations per year per doc	1.969
References	87042
**Document types**	
article	4176
article; data paper	1
article; early access	65
article; proceedings paper	73
article; publication with expression of concern	1
**Document contents**	
Keywords Plus (ID)	6021
Author's Keywords (DE)	5721
**Authors**	
Authors	19158
Author Appearances	36899
Authors of single-authored documents	27
Authors of multi-authored documents	19131
**Authors collaboration**	
Single-authored documents	31
Documents per author	0.225
Authors per document	4.44
Co-authors per documents	8.55
Collaboration index	4.46

**Figure 1:**
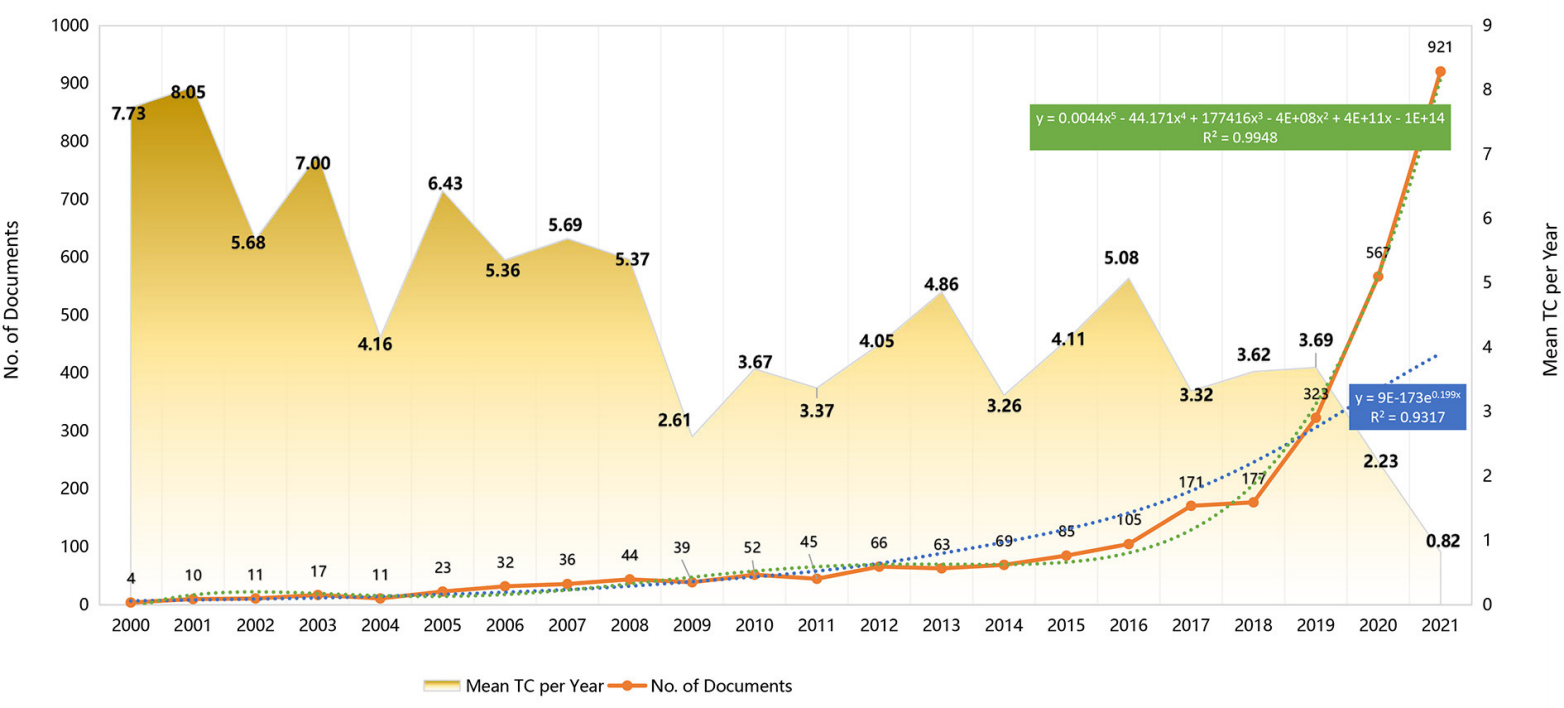
Year-wise publications and total citations (2000–2021). The growth of nomogram publications of original article conforms to the fitting curve, y = 0.0044x5 – 44.171x4 + 177416x3 – 4E+08x2 + 4E+11x – 1E+14, R^2^ = 0.9948. TC, Total citations.

Figure 1 shows annual production and citations of nomogram publications. Production is limited at first, but increases over time. Before 2018, the average annual growth rate was 23.6%; however, the past 3 years witnessed a surge past the exponential growth rate. The overall growth curve can be fitted into the fifth-order equation y = 0.0036x^5^ − 0.157x^4^ + 2.3614x^3^ − 14.069x^2^ + 30.529x, R^2^ = 0.994. It is estimated that the number of publications in 2022 will be between 648 and 1,222. In the first decade (2001–2010), the average total citations per year were higher, reaching a maximum in 2001.

A total of 74 countries and regions have contributed to this article. Table 2 shows the top 10 most productive countries based on their affiliation of the corresponding authors. China accounted for the largest articles published (8,829, 25.0%), followed by the United States (2,597, 14.78%). However, total citations and average article citations were highest in the United States (26,176 and 48.21, respectively). China's total citations ranked second with 15,929; however, its average article citation was only 6.17, ranking 38^th^. Furthermore, Canada had the highest proportion of multiple country publications (59.46%) in total publications, indicating a high degree of inter-country collaboration among its publications. On the other hand, China ranked least at 7.2%.

**Table 2:** The top 10 most productive countries, based on the affiliation of the corresponding author in the original articles.

**Region**	**Number of articles (%)**	**Total publications (%)**	**Total citations**	**Average article citation**	**SCP**	**MCP**	**MCP/total ratio (%)**
China	2582 (59.88)	8829 (50.24)	15929	6.17	2396	186	7.20%
USA	543 (12.59)	2597 (14.78)	26176	48.21	413	130	23.94%
Italy	162 (3.76)	1066 (6.07)	4006	24.73	96	66	40.74%
Korea	154 (3.57)	646 (3.68)	2372	15.4	136	18	11.69%
France	104 (2.41)	749 (4.26)	1884	18.12	73	31	29.81%
Japan	101 (2.34)	458 (2.61)	1682	16.65	88	13	12.87%
Canada	74 (1.72)	350 (1.99)	2980	40.27	30	44	59.46%
Netherlands	73 (1.69)	331 (1.88)	2509	34.37	37	36	49.32%
Germany	58 (1.35)	422 (2.40)	1524	26.28	26	32	55.17%
Spain	52 (1.21)	324 (1.84)	792	15.23	43	9	17.31%

SCP, single country publications; MCP, multiple country publications.

Multiple country publication ratio was calculated as MCP divided by the total of published documents per country.

**Figure 2:**
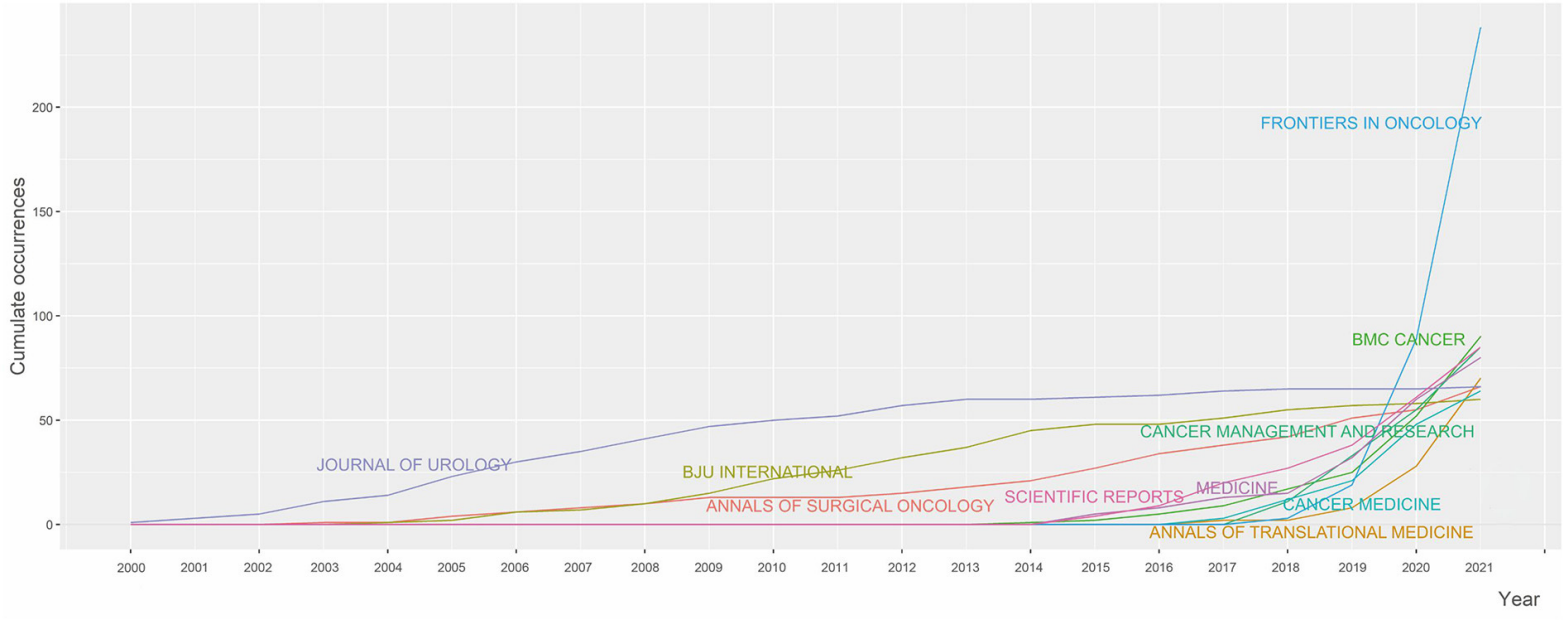
Top 10 source growth.

The retrieved articles were published in 138 different sources, with 1,411 published on Sources in Zone 1. The top 10 sources published 910 articles (31.7%), among which 5 journals pertained to oncology, with 549 articles (9.12%) published (Table 3). The journal with the largest number of papers was Frontiers in Oncology (*n* = 238), while Journal of Urology had the highest H-index (41), with a total citation of 5,790. Figure 2 shows the publication trends of the top 10 journals over time. A smoothing line was drawn using regression analysis based on the Loess Smoothing technique. The most significant curve was the continuous increase in the number of articles published in the Journal of Urology, while the number of articles published in Frontiers in Oncology rapidly increased in the last 3 years.

**Table 3:** Top 10 most productive sources.

**Sources**	**Number of articles (%)**	**h-index**	**g-index**	**m-index**	**PY_start**	**Zone**	**Journal impact factor (2021–2022)**
Frontiers in Oncology	238 (8.29)	12	19	2.4	2018	Zone 1	5.738
BMC Cancer	90 (3.13)	11	17	1.222222	2014	Zone 1	4.638
Cancer Management and Research	85 (2.96)	10	13	2	2018	Zone 1	3.602
Scientific Reports	85 (2.96)	15	21	1.875	2015	Zone 1	4.996
Medicine	80 (2.79)	11	14	1.375	2015	Zone 1	1.817
Annals of Surgical Oncology	70 (2.44)	25	47	1.25	2003	Zone 1	4.339
Annals of Translational Medicine	70 (2.44)	7	14	1.166667	2017	Zone 1	3.616
Cancer Medicine	66 (2.30)	12	17	2	2017	Zone 1	4.711
Journal of Urology	66 (2.30)	41	66	1.782609	2000	Zone 1	7.600
BJU International	60 (2.09)	25	43	1.315789	2004	Zone 1	5.969

**Figure 3:**
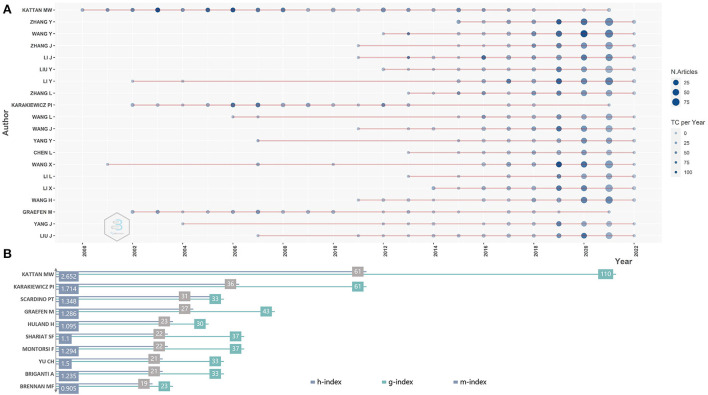
**(A)** Top 20 authors with nomogram publications in the research period. **(B)** Top 10 authors with local impact by H-index.

Figure 3A shows a diagram of the top 20 most prolific nomogram authors during the study period. The size of the dots represents the number of articles, and the strength of the colors represents the total number of citations per year. In terms of the number of articles published during the study period, the top three most prolific authors were Kattan MW (134 articles), Zhang Y (87 original articles), and Zhang J (80 original articles). The top 10 authors' local impact by H-index are shown in Figure 3B.

According to the number of citations during the study period, the top 10 original articles are shown in Table 4. The top globally cited article was published by Iasonos A in 2008 on the build and interpretation of the nomogram for cancer prognosis. The second and third articles were Huang YQ, 2016, J Clin Oncol and Wang YZ, 2013, J Clin Oncol, on tumor radiomics and tumor prognosis, ranking third and second in local citations, respectively. Excluding Van Zee KJ, 2003, Ann Surg Oncol, the remaining 9 articles were published in J Clin Oncol. There were 2,190 articles (52.44%) that were not cited; the median and average citations per article were 4 and 15.44, respectively.

**Table 4:** The top 10 original articles based on citations in the research period.

**Rank**	**First author**	**Title**	**Journal**	**Year of publication**	**Local citations**	**Global citations**	**LC/GC ratio (%)**
1	Iasonos A	How to build and interpret a nomogram for cancer prognosis	J Clin Oncol	2008	584	1168	50.00
2	Wang YZ	Prognostic nomogram for intrahepatic cholangiocarcinoma after partial hepatectomy	J Clin Oncol	2013	228	616	37.01
3	Liang WH	Development and validation of a nomogram for predicting survival in patients with resected non-small-cell lung cancer	J Clin Oncol	2015	179	320	55.94
4	Huang YQ	Development and Validation of a Radiomics Nomogram for Preoperative Prediction of Lymph Node Metastasis in Colorectal Cancer	J Clin Oncol	2016	174	769	22.63
5	Han DS	Nomogram predicting long-term survival after d2 gastrectomy for gastric cancer	J Clin Oncol	2012	112	219	51.14
6	Van Zee KJ	A nomogram for predicting the likelihood of additional nodal metastases in breast cancer patients with a positive sentinel node biopsy	Ann Surg Oncol	2003	102	597	17.09
7	KattanMW	Postoperative nomogram for disease-specific survival after an R0 resection for gastric carcinoma	J Clin Oncol	2003	91	320	28.44
8	Karakiewicz PI	Multi-institutional validation of a new renal cancer-specific survival nomogram	J Clin Oncol	2007	85	382	22.25
9	Bochner BH	Postoperative nomogram predicting risk of recurrence after radical cystectomy for bladder cancer	J Clin Oncol	2006	82	339	24.19
10	Katttan MW	Postoperative nomogram for 12-year sarcoma-specific death	J Clin Oncol	2002	76	393	19.34

LC, Local Citations; GC, Global Citations.

**Figure 4:**
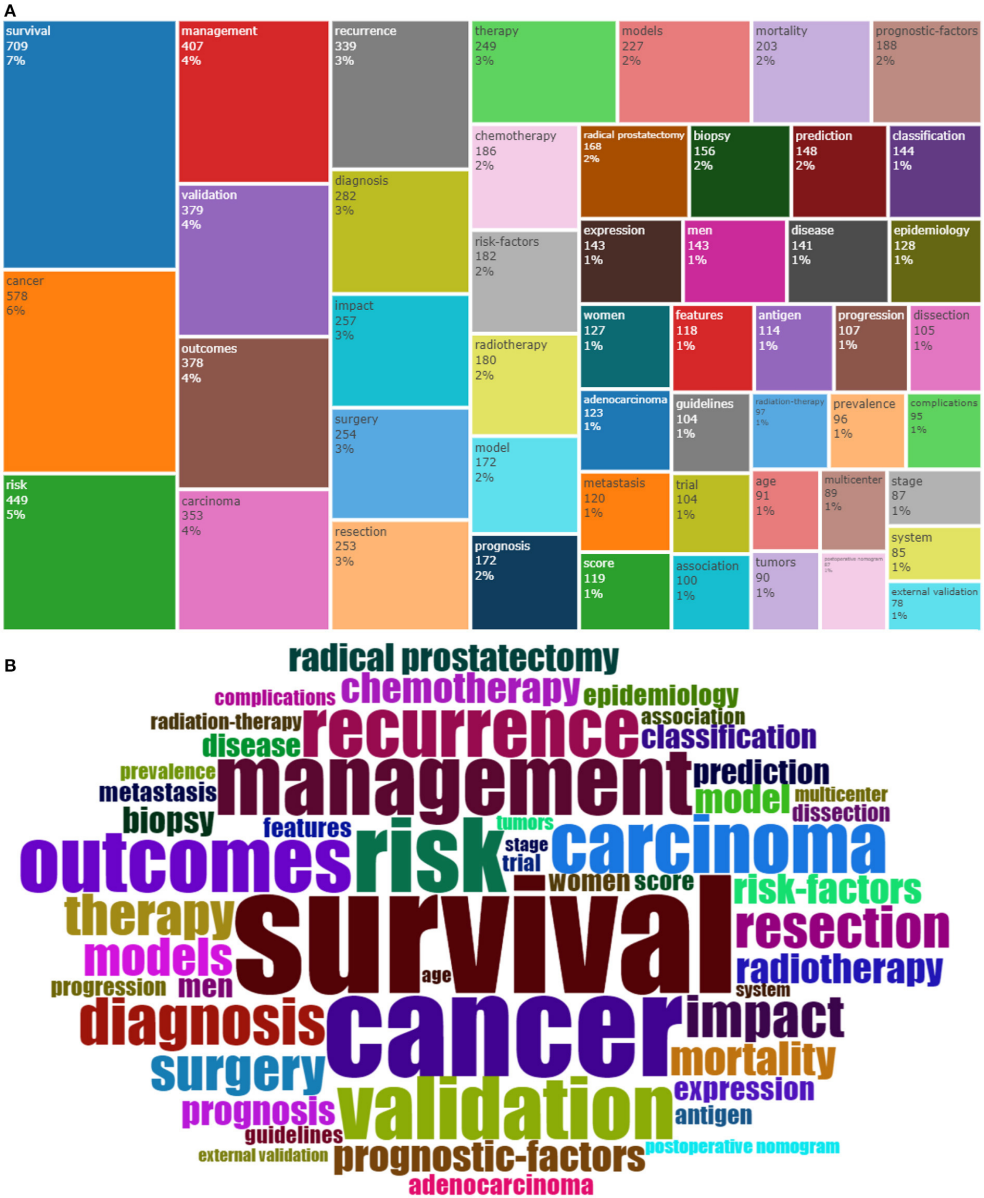
**(A)** Treemap chart of the 50 most frequently occurring “KeyWords Plus” terms. **(B)** World cloud of the 50 most frequently occurring “KeyWords Plus” terms.

A total of 37,321 keywords were included in the keywords Plus list. Figure 4A shows a tree of the 50 most frequently used terms based on “Keywords Plus”. A larger rectangular area represents a larger proportion of a particular term. Survival, cancer, management, validation, and outcomes are the five most prominent terms. “Keywords Plus” generates word cloud, as shown in Figure 4B.

**Figure 5:**
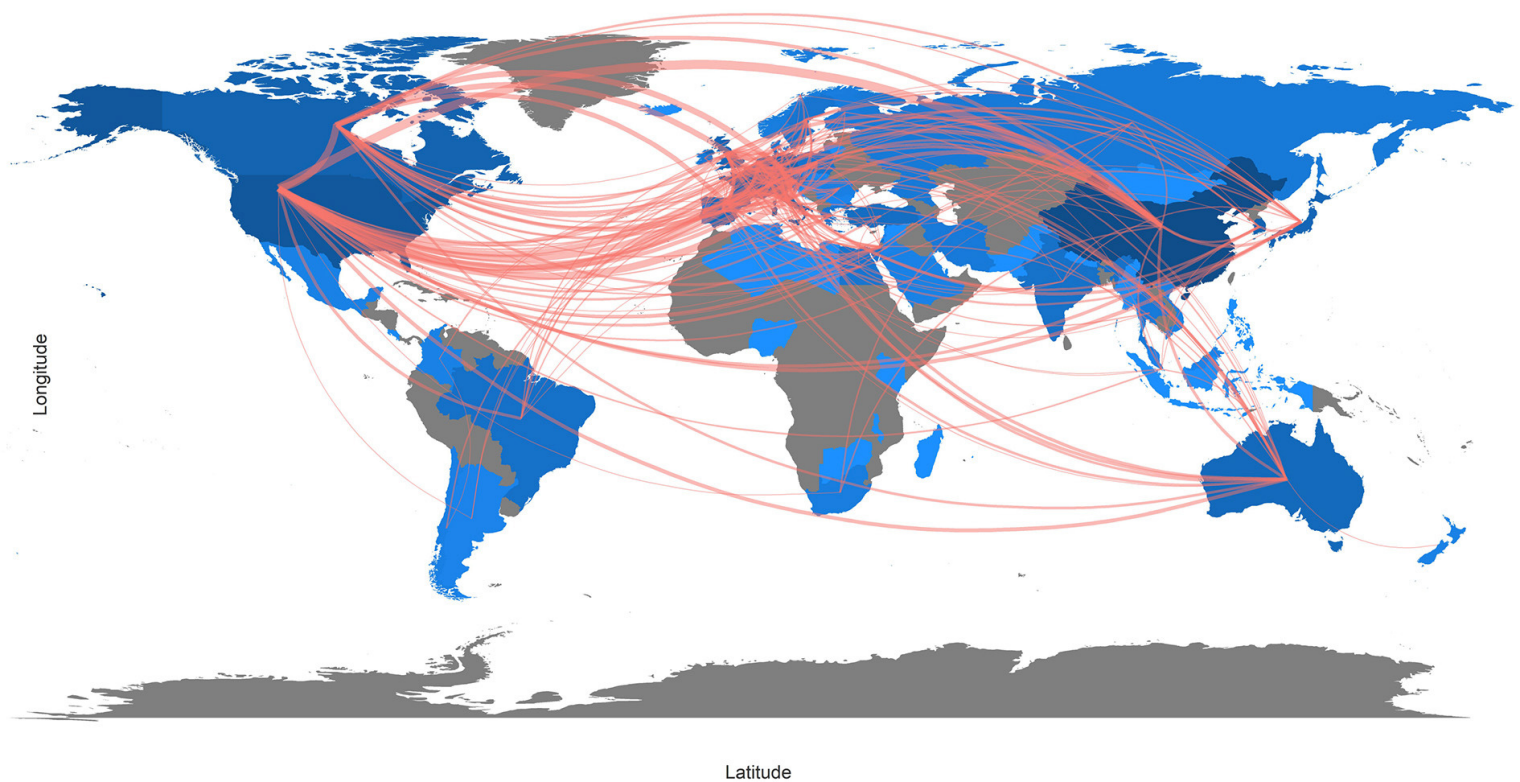
Country Collaboration Map of nomogram original articles.

The international research collaboration for original nomogram articles is shown in Figure 5. The number of published articles is indicated by the intensity of the blue color. The thickness of the red line indicates the intensity of frequency-based collaboration. The United States had the strongest cooperation with other countries, followed by Canada (Frequency = 87), Germany (Frequency = 64), France (frequency = 40), Netherlands (Frequency = 38), United Kingdom (Frequency = 26), Austria (Frequency = 23) and Australia (Frequency =21). The second most cooperative country was Italy, including Germany (Frequency = 60), Canada (Frequency = 57) and France (Frequency = 46).

**Figure 6:**
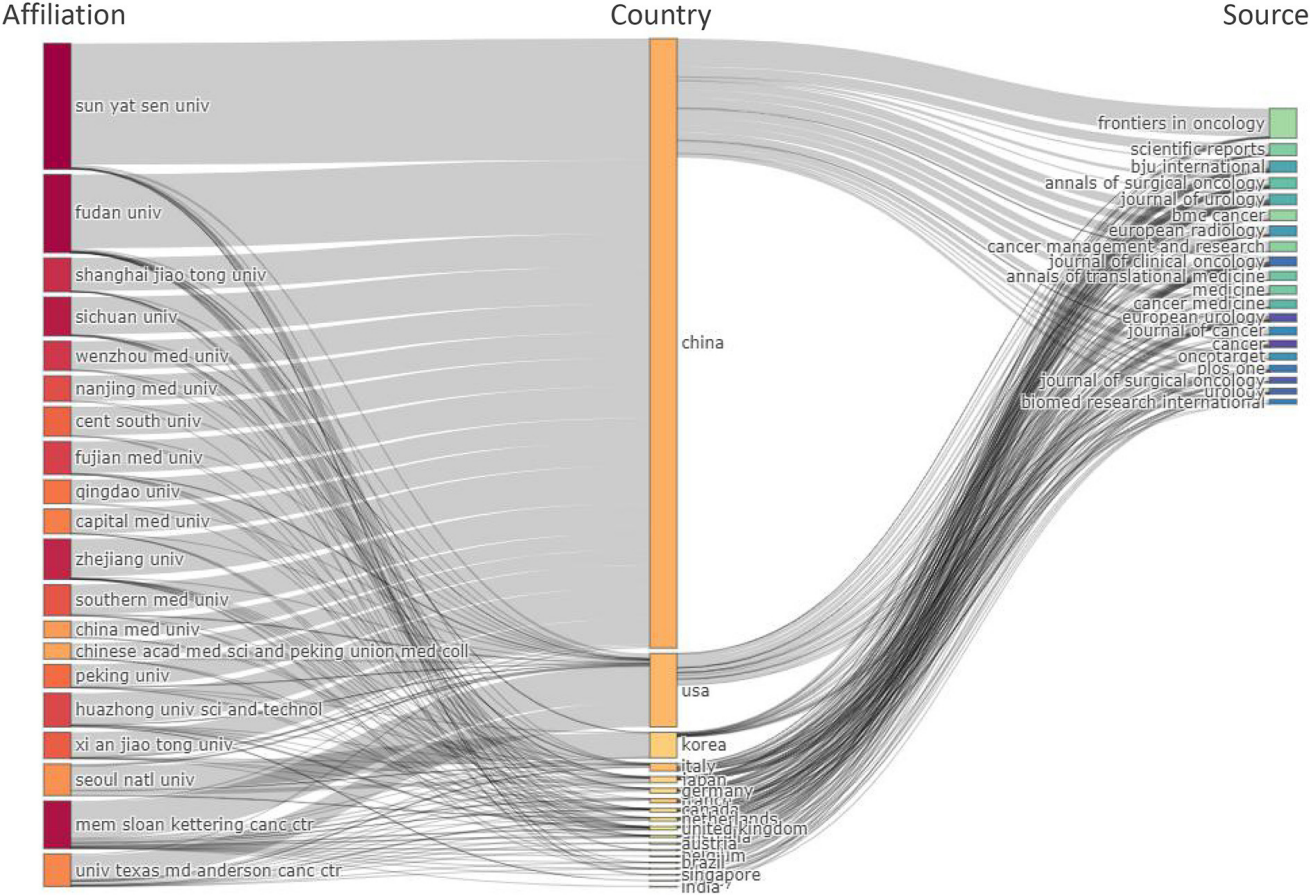
Three-field plot showing the network between institutions (left), countries (middle), and journals (right).

Figure 6 shows a three-field plot based on the Sankey chart, which describes the relationship between countries, institutions, and journals. The height of the rectangular nodes is proportional to the frequency of the presence of a particular country, institution or journal in the collaborative network. The width of the lines between nodes is proportional to the number of connections. The results show China as the dominant country (Frequency =17,152), comprising 17 of the top 20 institutions. For example, Sun Yat Sen University (frequency = 3,406), Fudan University (frequency = 2,183) and Zhejiang University (frequency = 1,126) were included. It was followed by the United States (frequency = 2,064) and South Korea (frequency = 713). Besides China, the top contributing institutions were Memorial Sloan Kettering Cancer Center (1,334) and MD Anderson Cancer Center (911), both from the US, followed by Seoul National University (832) from South Korea. In the journal on the right, the leading contributors to Frontiers in Oncology were China (768/832 = 92.31%), followed by the United States (12/832 = 1.44%) and Italy (12/832 = 1.44%). In the Journal of Urology, the main contributor with the highest H-index was the United States (147/299 = 48.16%).

**Figure 7:**
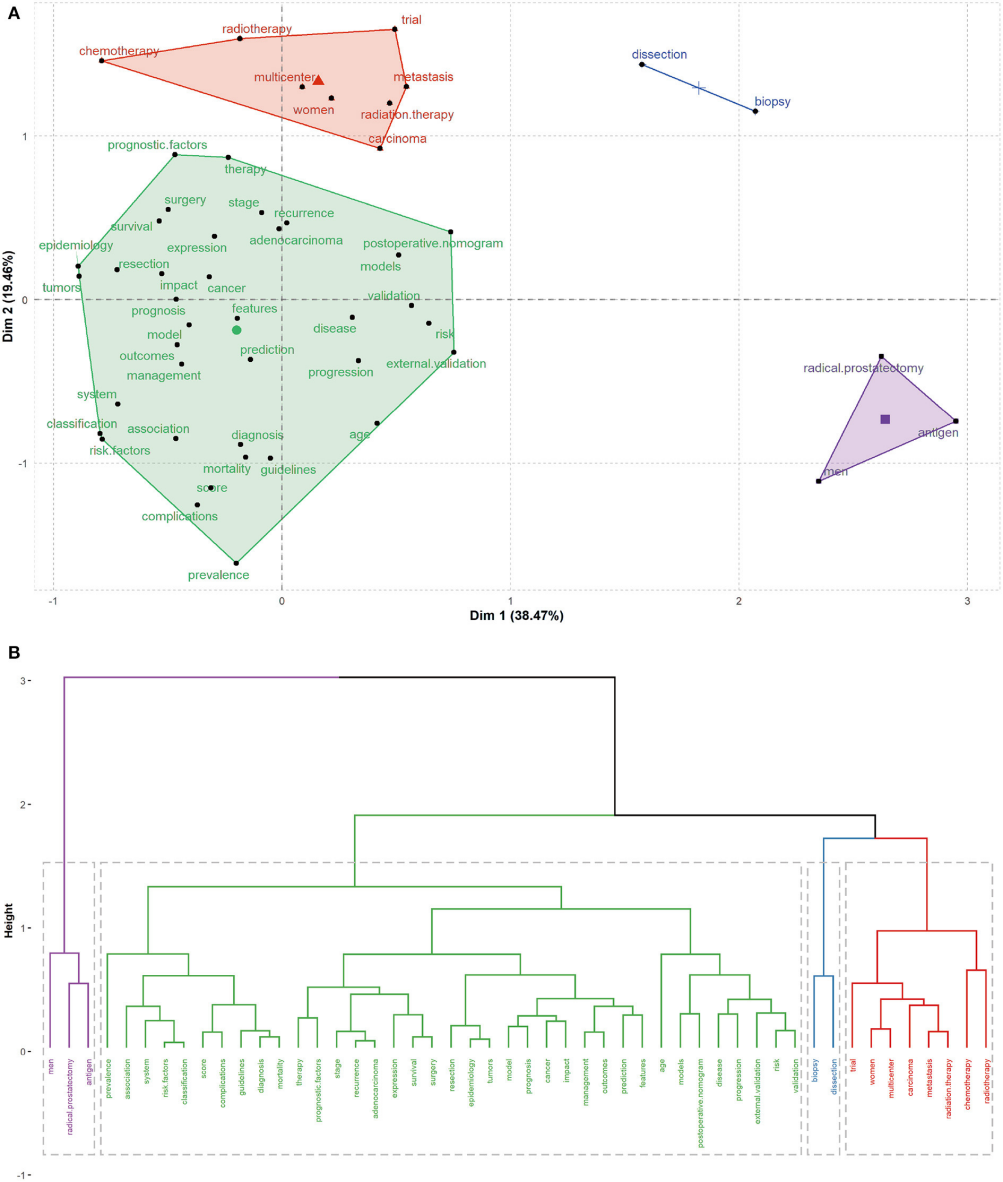
**(A)** Multiple correspondence analysis of high-frequency keywords in nomogram original articles. The figure shows that the cluster closest to the center is the core cluster. **(B)** Dendrogram of hierarchical cluster analysis of author key words. The entire classification structure forms a tree dendrogram, displaying the close association between the keywords in the field of nomogram.

**Figure 8:**
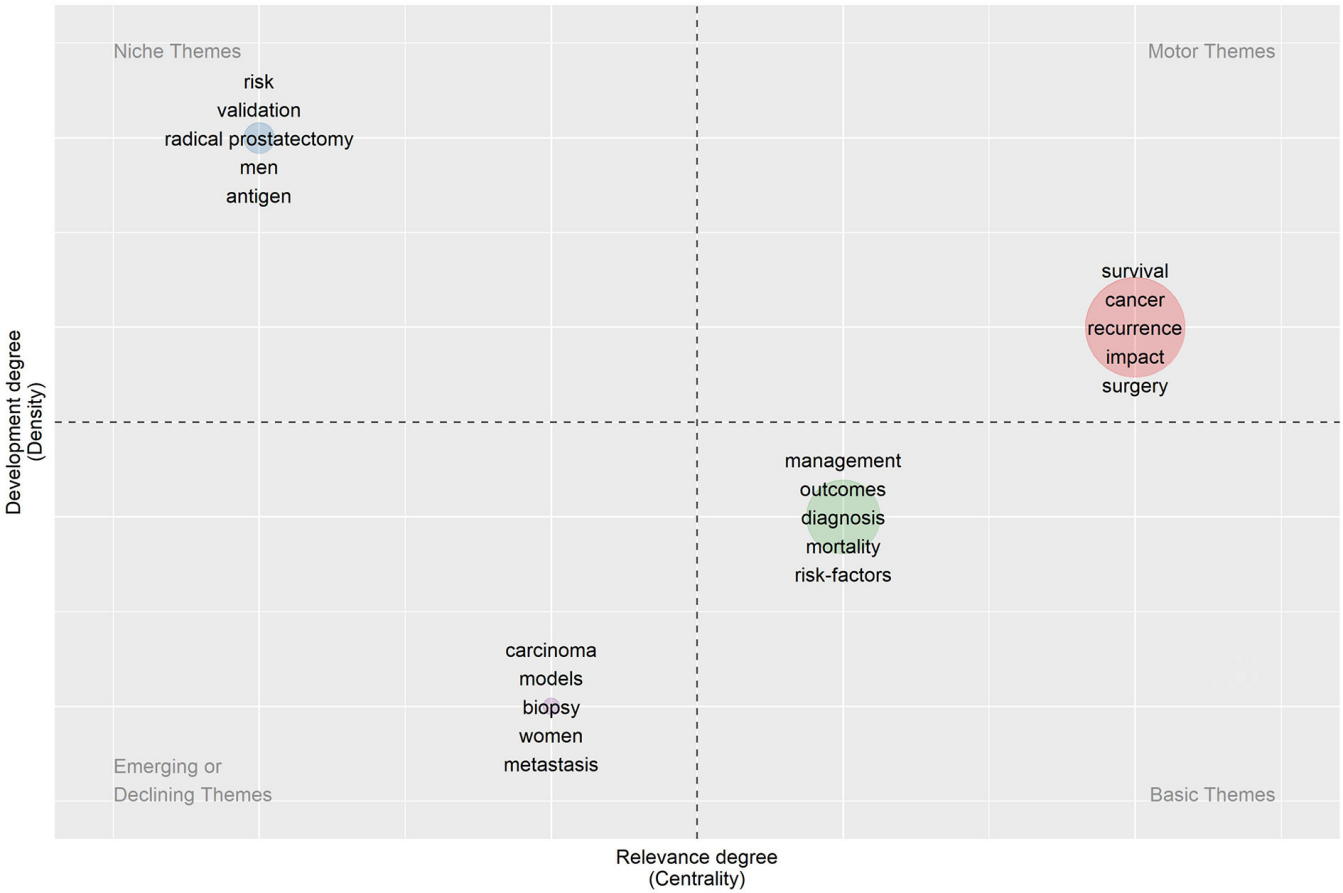
Thematic map based on keywords plus.

A final analysis of the nomogram's published data records will be conducted using MCA to discover relevant keywords and their synonyms, and evaluate their relationships to study how different concepts relate to each other in the study (Figure 7). For the nomogram subject clustering, four categories are represented as Cluster 1 (red), cluster 2 (blue), cluster 3 (green), and Cluster 4 (Purple). Cluster 1: Adjuvant therapy of tumors, including radiotherapy and chemotherapy; Cluster 2: biopsy, dissection; Cluster 3: prognosis, recurrence, survival, surgery, outcome, prediction, and epidemiological indicators of the tumor, as well as specific nomogram indicators, including risk factors, score, classification, diagnosis, complications, guidelines, and mortality. Cluster 4: Male prostate tumors and their antigen detection. As seen from the thematic map, cluster 3 has become a mature research topic, and some of its research topics are located in the fourth quadrant, representing a better developmental direction. Clusters 2 and 4 are located in the second and fourth quadrants, respectively (Figure 8).

**Figure 9:**
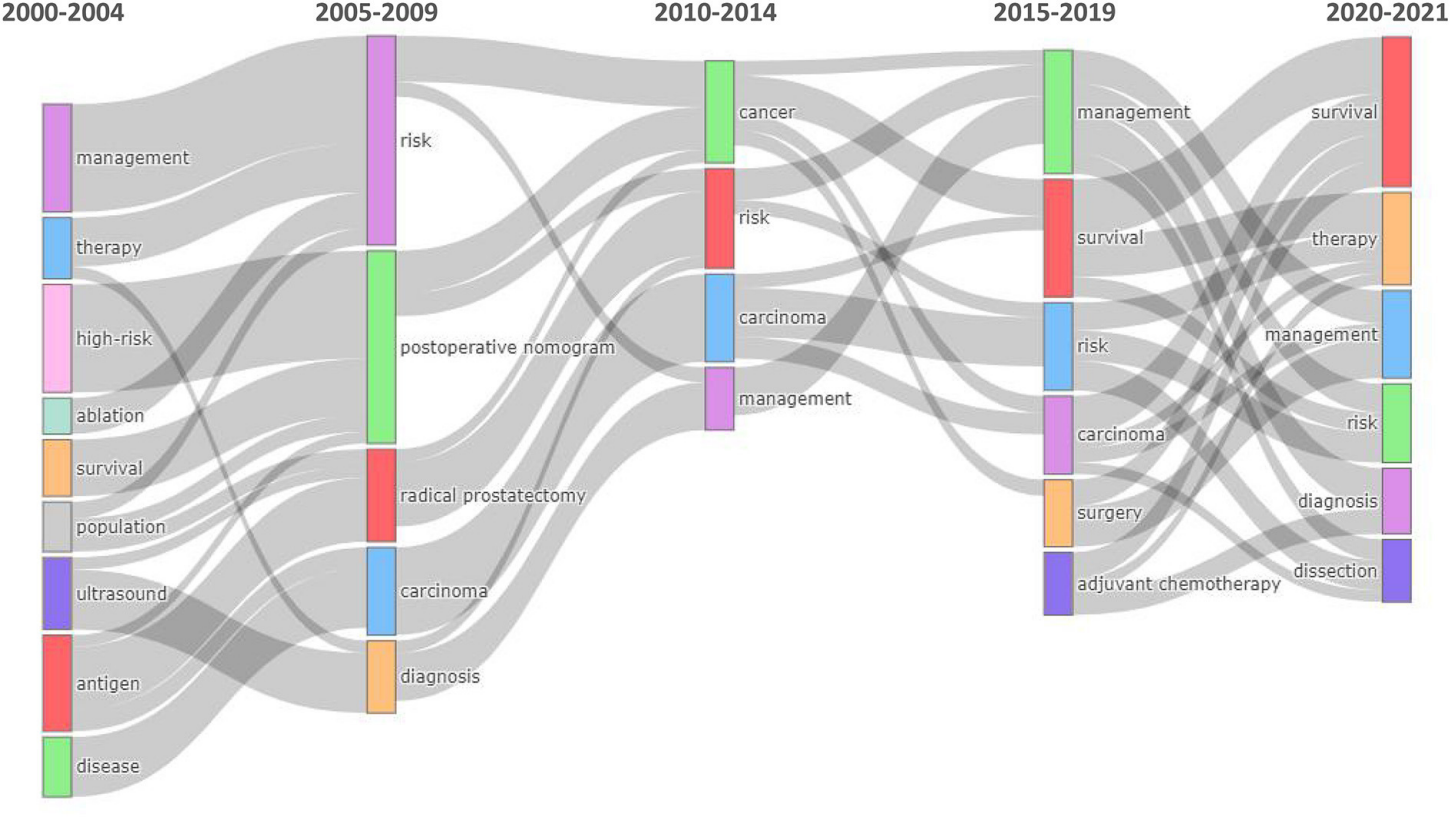
Thematic evolution of keywords in the field of research on nomogram from 2000 to 2021.

In the theme's evolution, the 20-year period was divided into five stages: 2000–2004, 2005–2009, 2010–2014, 2015–2019, and 2020–2021 (Figure 9). The first stage was the initial stage of nomogram research, with few keywords and loose research topics. Following the second stage, targeted studies have been conducted on tumors, especially urinary system tumors and prognosis. In the third and fourth stages, in-depth studies were conducted on tumor risk factors, prognosis, and management. With the development of nomogram studies and the number of articles published in recent years, no cancer or carcinoma has been identified. This suggests that the number of nomogram articles has increased over time, and the topic has evolved through further research in oncology into predicting diagnosis and treatment outcomes in other areas of medicine.

## Discussion

Since their creation in 1980, researchers have debated on whether nomogram techniques should be referred to as “nomographs” or “nomograms.” In scientometrics, this nomogram was first identified in 2000, and 4,176 original articles had been published by the end of 2021. In particular, the number of articles published every year has doubled since 2019, which may reflect the extensive promotion of nomogram and its multi-disciplinary use. This study shows that the scientific yields calculated by Lotka's Law have significant statistical similarity (*P* < 0.05). Therefore, we can infer that nomograms will continue to be of interest and the number of publications is likely to grow in coming years.

However, the growth in nomogram publications seems to be primarily driven by developing rather than developed countries. In terms of number of publications, China published the largest number of studies on the application of nomogram, especially in the past 5 years, which have played a key role in this field. In terms of publication sources, Frontiers in Oncology and BMC Cancer had the most publications, with 33.79% of records (1,411 documents) published in the Core of the Bradford Zone. Both journals discuss tumor research, indicating that nomogram publications have a broader discussion on tumors. Deserving special attention is Frontiers in Oncology as the recent 3 years saw an increase in the number of studies on nomogram, and most of the studies were from China.

Nomogram was first used in medicine to predict the prognosis of diseases in oncology. Various risk factors of tumor and tumor occurrence were transformed into a quantitative statistical model, which can be used to screen high-risk groups and evaluate their risk of disease. This nomogram could provide statistical evidence to support clinical diagnosis of cancer in its probabilistic prediction of cancer risk. The National Cancer institute has collected many kinds of cancer risk prediction model (14) including breast cancer risk assessment nomogram (http://www.cancer.gov/bcrisktool); it included personal and family history of breast cancer, to build a statistical model and calculate the risk of invasive breast cancer, in order to effectively predict breast cancer incidence. The nomogram based on the BOADICEA (Breast and Ovarian Analysis of Disease Incidence and Carrier Estimation Algorithm) prediction model can be used to predict breast or ovarian cancer risk in individuals with BRCA1 and BRCA2 mutations (15). Timmers et al. (16) analyzed and established a nomogram for breast cancer risk prediction by collecting 352 mammography and pathological data, and using multivariate logistic regression to help Radiology doctors to determine breast cancer risk by imaging results.

Another important application of this nomogram in cancer would be to evaluate the prognosis of tumors and provide a reference for clinicians to select treatment options for patients. This research hotspot both develops and forms an important pillar that shapes the research field in the thematic map. Liang WH constructed a nomogram based on the clinical data of 6,111 patients with non-small cell lung cancer (NSCLC) in China to identify 6 independent prognostic factors, including gender, age, and pathological type (17). Nomogram has a better prognostic assessment of lung cancer than the Joint Commission on Cancer TNM Staging (7th Edition). In 2008, Weiser incorporated clinicopathological variables to establish a nomograph that could be used to predict recurrence in patients with colorectal cancer after surgery (18). After that, Ying constructed a nomogram by increasing the preoperative high neutrophils-to-lymphocytes (NLR) variable, which was used to evaluate recurrence-free survival, overall survival, and cancer-specific survival of colorectal cancer patients (19). Furthermore, studies are available on incorporating microRNA into the construction of nomogram (20).

In the research of oncology, the research direction of urinary tumor is the most mature, and it can even be clustered separately in this study. This cluster is located in the upper left quadrant, with higher values of density but lower values of centrality that reflect the highly developed but isolated themes. Since the late 1990s, several models have been developed to help physicians diagnose and manage prostate cancer (21, 22). The model for predicting lymph node invasion in patients with radical prostatectomy may represent the most popular rograph used in urological clinical practice (23). They help physicists and urologists distinguish between patients who should undergo pelvic lymph node dissection during radical prostatectomy and those who should not (24–27). Since the establishment of this nomogram, Cagiannos, Godoy, Memorial Sloan Kettering Cancer Center (MSKCC) and Briganti nomograms have repeatedly demonstrated their validity and accuracy (28–31). All studies relied on clinical variables, such as baseline prostate-specific antigen (PSA) value, clinical T staging, and the biopsy Gleason score.

Over time, nomogram is no longer limited to the study of oncology, but more applied to the study of diagnosis and prediction of non-tumor-related diseases. For example, nomograms were used to analyze the correlation between factors in genomics or proteomics and the development and progression of diseases (32–34); Nomograms were used to predict adverse outcomes in obstetrics or reproductive medicine (35–38); it can also be combined with radiomics to analyze the relationship between different image features or image segmentation factors and disease diagnosis (39, 40), predict chronic disease incidence in orthopedics (41–43), or analyze and detect the incidence of chronic diseases combined with metabolomics (37). In addition, the display modes of nomogram are gradually diversified. In addition to pictures or tables, there are also web calculators or scoring systems based on clinical prediction models.

Of course, no matter what research direction nomograms are developed and established, multi-dimensional verification and evaluation are needed to verify the relationship between data and modeling data, and to clarify the effectiveness of nomograms. Data validation can be divided into internal validation and external validation, both of which aim to test the repeatability and portability of the prediction model. In addition, the evaluation indexes of the nomogram also include the discrimination and calibration. The discrimination degree is the ability to distinguish whether the end event occurs, which can be expressed by the objective index C statistic. The closer the C statistic is to 1, the better the discrimination degree of the model will be, while the C statistic is equal to 0.5, the model has no predictive ability. When the end point event is a dichotomous variable, the C statistic is equal to the area under the ROC curve (AUC). Calibration is used to evaluate the degree of agreement between the predicted probability of an event in a clinical prediction model and the observed objective probability of an event. When the calibration degree is poor, it indicates that the model has a large error in judging the risk possibility of the event. The most common display method is to draw the calibration curve, and the higher the fit between the fitted curve and the standard curve, the better the calibration of the model. On this basis, it is also necessary to evaluate the application value of the nomogram in medical research and the degree of individual benefit. For example, Dr. Andrew Vickers of Memorial Sloan Kettering Cancer Center developed a method called Decision Curve Analysis (DCA) in 2006 (44). There are two reference lines in DCA, one reflects the net benefit without any treatment, and the other is the net benefit of all patients receiving treatment. When the net benefit of the curve is higher than the two reference lines, it indicates that it has certain clinical application value.

The application of nomograms is a hot topic in clinical research; however, its characteristics may limit its widespread clinical application. First, its hypothesis is that the results will remain constant over time;; however, actual clinical practice limits its ability to predict real-time outcomes due to improved therapies, improved early detection, and natural disease variability (45). Furthermore, its performance lacks accepted reporting standards and can be highly variable. Some publications only reported discrimination without calibration, or often did not have confidence intervals or were not fully displayed (46, 47). This makes it impossible to assess its accuracy using different estimated probabilities. In the future, data mining and machine learning based on big data will improve the ability to predict diseases and gradually change the medical decision-making process (48). However, over-fitting in the process of machine learning or deep learning limits its clinical applicability, and the relationship between risk factors and outcomes is difficult for patients to understand. Therefore, nomograms can still be used in molecular biological, radiography and clinical indicators to predict diagnostic/prognostic outcomes for a long time.

This study is the first bibliometric analysis of nomograms. In recent years, bibliometric has gradually developed in medical research compared with the traditional systematic review (49). It can sort out the hot spots and evolution direction of medical research from the perspective of time and clustering, and also can educate medical students to avoid past mistakes (50) and understand the great achievements of discovery and progress. However, bibliometric analysis is limited, such as limited access to data published in the most complete form or limited database selection. Therefore, WOS was selected for bibliometric analysis as each indexed article had complete and indexed research data, such as author, source, cited references, keywords, and research field. This study does not critically analyze the content of each paper, but only describes the research trend of this topic. In future, we will consider other data sources, such as Scopus and PubMed data, to determine future trends.

## Conclusion

This study shows the general trend of Nomogram in scientific research. The number of published studies has increased rapidly since 2018 and is expected to increase in the coming years. In recent years, research on the application of nomogram has been developing among non-oncology subjects, and nomogram will be applied to predict the relationship between genomics, proteomics, radiomics and outcomes, relying on the development of big data and bioinformatics in the future. In this regard, bibliometrics will continue help identify hotspots and gaps for future research by providing comprehensive analysis and structured information on this topic.

## Data availability statement

The original contributions presented in the study are included in the article/supplementary material, further inquiries can be directed to the corresponding authors.

## Ethics statement

The study is based on a public database and does not require Ethics Committee approval. Informed consent was obtained from all individual participants included in the study.

## Author contributions

XW and DZ designed the study and drafted the manuscript. JL and ZS determined the strategy. YZ designed the statistical analysis plan. TL and DZ reviewed the manuscript. All authors take responsibility for the appropriateness of the content. All authors contributed to the article and approved the submitted version.

## Funding

This work was supported by internal funding from Shengjing Hospital, China Medical University (SJ-M0133), 345 Talent Project of Shengjing Hospital of China Medical University (No.: M0946), and Medical Education Research Project of Liaoning Province (No. 2022-N005-03).

## Conflict of interest

The authors declare that the research was conducted in the absence of any commercial or financial relationships that could be construed as a potential conflict of interest.

## Publisher's note

All claims expressed in this article are solely those of the authors and do not necessarily represent those of their affiliated organizations, or those of the publisher, the editors and the reviewers. Any product that may be evaluated in this article, or claim that may be made by its manufacturer, is not guaranteed or endorsed by the publisher.
